# Plasma-Induced Wafer-Scale Self-Assembly of Silver Nanoparticles and Application to Biochemical Sensing

**DOI:** 10.3390/ma8073806

**Published:** 2015-06-24

**Authors:** Yunbo Shi, Hao Guo, Jiangtao Yang, Miaomiao Zhao, Jun Liu, Chenyang Xue, Jun Tang

**Affiliations:** 1 Science and Technology on Electronic Test & Measurement Laboratory, North University of China, Taiyuan 030051, Shanxi, China; E-Mails: shiyunbo@nuc.edu.cn (Y.S.); guohaonuc@gmail.com (H.G.); yangjiangtaonuc@gmail.com (J.Y.); zhaomiaomiaonuc@gmail.com (M.Z.); liuj@nuc.edu.cn (J.L.); xuechenyang@nuc.edu.cn (C.X.); 2 Key Laboratory of Instrumentation Science & Dynamic Measurement (North University of China), Ministry of Education, Taiyuan 030051, Shanxi, China

**Keywords:** wafer-scale, plasma-induce, nanoparticles, self-assembly, biochemical sensing

## Abstract

In this work, the wafer-scale silver nanoparticles fabricated by a self-assembly method was demonstrated based on a magnetron sputtering and plasma treatment process. Silver nanoparticles of different sizes and shapes were prepared, and the effects of the plasma treatment time, plasma gas composition, and power were systematically investigated to develop a method for low-cost and large-scale fabrication of silver nanoparticles. Furthermore, the surface-enhanced Raman scattering experiments: crystal violet, as the probe, was absorbed on the silver nanoparticles film of different size and density, and get the phenomena of surface-enhanced Raman scattering and surface-enhanced fluorescence. The results show that the proposed technique provides a rapid method for the fabrication of silver nanomaterial; the method is adaptable to large-scale production and is compatible with the fabrication of other materials and biosensors.

## 1. Introduction

Due to the unique optical and electronic properties of the metallic nanoparticles, precious metal nanomaterials such as gold and silver have received considerable attention in recent years, leading to the development of increasingly sensitive analytical techniques with the advancement of nanotechnology [1,2]. Their potential applications span over many new fields, such as catalysis, photography, optics, electronics, optoelectronics, information storage, biological and chemical sensing, and surface-enhanced Raman scattering (SERS) [3,4,5,6].

Therefore, several approaches for the fabrication of silver nanomaterials, such as self-assembly of silver colloids, lithographic fabrication, vapor deposition, chemical deposition, ultraviolet (UV) irradiation, electron beam irradiation *etc.* have been developed [7,8,9,10,11,12].

The chemical methods adopted for the fabrication of silver nanomaterials require the addition of harsh reducing reagents, which may result in the presence of chemical residues after the reaction. The high surface energy of such particles makes them extremely reactive, and most systems undergo aggregation without an appropriate protection or passivation of their surface. Some passivating agents, such as surfactants and amine or thiol-functional organics, are commonly used to protect the nanoparticle surface [13,14]. Most of them are highly reactive chemicals and pose potential environmental and biological risks. Furthermore, the electron beam technique is not extensively used because the electron beam irradiation apparatus is expensive and hard to set on a laboratory scale.

Recently, the plasma-induced technique presents some distinct advantages in the preparation of nanomaterials, such as short reaction time, simple operation, and the absence of chemical residues after the reaction. It prospects a feasible way to synthesize nanomaterials in a wafer-scale.

This paper reports the synthesis of silver nanomaterials with different sizes and shapes on substrates using a plasma-induced technique. Ag nanoparticles were deposited on p-doped Si substrates using a direct-current (DC) magnetron sputtering process. By changing the plasma (O_2_ and/or Ar) treatment conditions (such as the plasma treatment time, plasma gas composition, and power), Ag nanomaterials of different sizes and patterns were formed. And this wafer-scale nanomaterials have been used as the SERS substrate to enhance the Raman signal for crystal-violet (CV) probe. Compared to other reported methods for the fabrication of silver nanomaterials, the proposed technique provides a rapid method that can be adapted to large-scale production and is compatible with the fabrication of other materials and biosensors.

## 2. Results and Discussion

### 2.1. Preparation of Wafer-Level Low-Dimensional Structures

**Figure 1:**
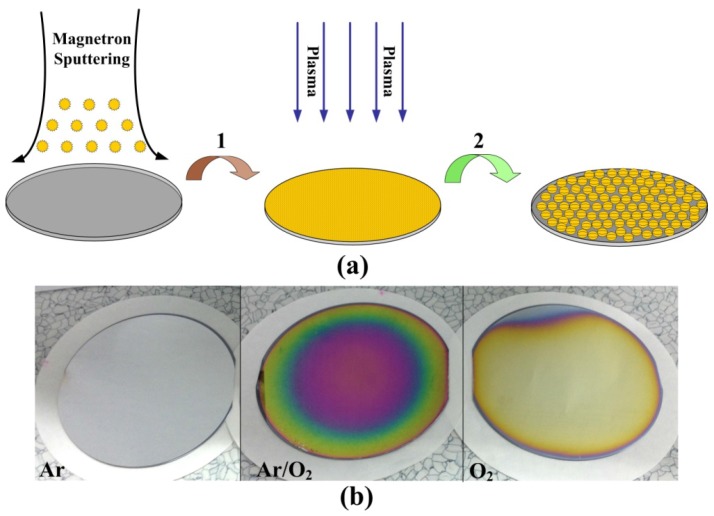
(**a**) Schematic illustration of the fabrication process of the silver nanomaterials; (**b**) The wafer-scale silver nanomaterials fabricated by the Argon and/or Oxygen atmosphere plasma technology.

Figure 1 shows the two steps of the overall process for the fabrication of silver nanomaterials of various sizes and shapes on the substrates: (a) preparation of the homogeneous silver films and fabrication of silver nanomaterials by the plasma-induced technique; (b) preparation of a 3 inch wafer-level nano-structure with argon gas, a mixed gas of argon and oxygen, and oxygen. The homogeneous silver films were prepared on p-type silicon substrates using the DC magnetron sputtering technique. The plasma-induced synthesis was achieved using an inductively coupled plasma etching system.

### 2.2. Ordered Controlled Preparation of Low-Dimensional Structures

**Figure 2:**
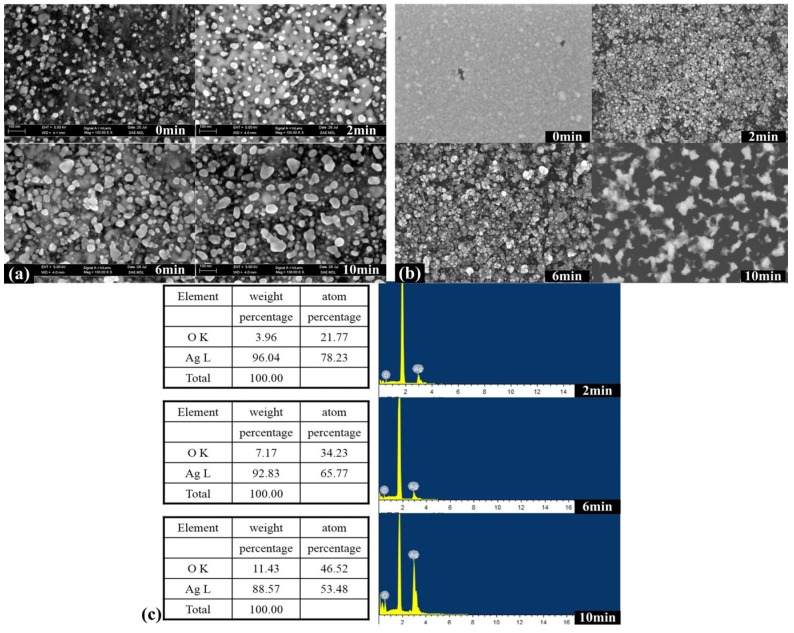
Controlled preparation of nanoparticles by argon or oxygen atmosphere plasma technology under different treatment time (2 min, 6 min, and 10 min) in the power of 120 W. (**a**) By Ar atmosphere; (**b**,**c**) By oxygen atmosphere and the corresponding energy spectrum.

Figure 2 shows the controlled preparation of ordered nanoparticles by argon or oxygen atmosphere plasma technology. The plasma power is 120 W and the treatment time is 2, 6 and 10 min respectively with an O_2_:Ar ratio of 0%:100% or 100%:0%. As shown in Figure 2a, from the images of the silver nanomaterials which were characterized with scanning electron microscope (SEM), as the treatment time increases, the nanoparticle size becomes larger with an O_2_:Ar ratio of 0%:100%.

Figure 2b shows the changes of the size and morphology for the silver nanostructures treated by the oxygen atmosphere. With the increase of treatment time, the morphology of silver nanomaterials changes from the isolated structure to the pore structure gradually with an O_2_:Ar ratio of 100%:0%. Moreover, as shown in Figure 2c, the corresponding energy spectrum shows the coincident results. As the treatment time increases, the more concentration of oxygen can be testing in the nanoparticles, due to the more oxygen have been reacted with the Ag atom.

The results obtained for the samples synthesized under pure oxygen are very different from pure argon. In the argon atmosphere environment, the diffusion of silver atoms will be rather slow because of the high energy barrier required to break the Ag–Ag bonds [15,16]. The oxidation of Ag nanoparticles greatly reduces the energy barrier to break the Ag–Ag bonds. This analysis is consistent with the experimental results shown in Figure 2c. Thermodynamically, silver can form silver oxide (Ag_2_O) by reaction with oxygen in the present experimental conditions. The oxygen content increases significantly in the nanoparticle composition, and the particles gradually change from Ag_2_O to AgO. Thus, by controlling the gas environment in the synthesis process, it is a potential method to synthesize the oxide, nitride, fluoride nanoparticle/nanostructure, and so on.

**Figure 3:**
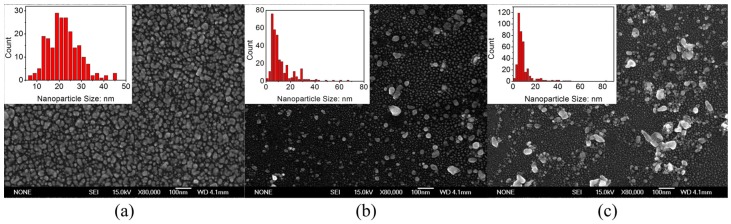
Scanning electron microscopy images of silver nanomaterials fabricated at different plasma power: (**a**) 5 W; (**b**) 10 W; (**c**) 20 W; under the O_2_:Ar ratio is 25%:75%.

The power evolution of the plasma treatment of silver nanomaterials deposited on silicon substrates was studied. The conditions are as follows: the plasma treatment time was 2 min; the power values in Figure 3a–c are 5, 10 and 20 W, respectively, with an O_2_:Ar ratio of 25%:75%. After different durations of the plasma treatment, the silver nanomaterials were characterized by SEM, as shown in Figure 3. From the SEM analysis, the nanomaterials seem to aggregate and assume a larger size as the plasma power increases. However, the mean particle size clearly decreases and the particle density increases with increasing plasma power. However, the particles are induced toward be polarized as the larger or smaller and the mean size of the nanoparticles decreased from 21.6 ± 2 nm at 5 W of plasma power to 11.4 ± 2 nm at 10 W, and to 8.7 ± 1 nm at 20 W. Simultaneously, the density increased from 5.46 × 10^10^ to 8.06 × 10^10^, and then 9.78 × 10^10^ cm^−2^, respectively. Furthermore, as the plasma treatment power increases, the particles assume both larger and smaller sizes. In other words, at higher plasma energy, larger and smaller particles may be produced at the same time. Our experiments involve multiple types of diffusion and chemical reactions.

### 2.3. Wafer-Scale Low-Dimensional Ordered Structures 

**Figure 4:**
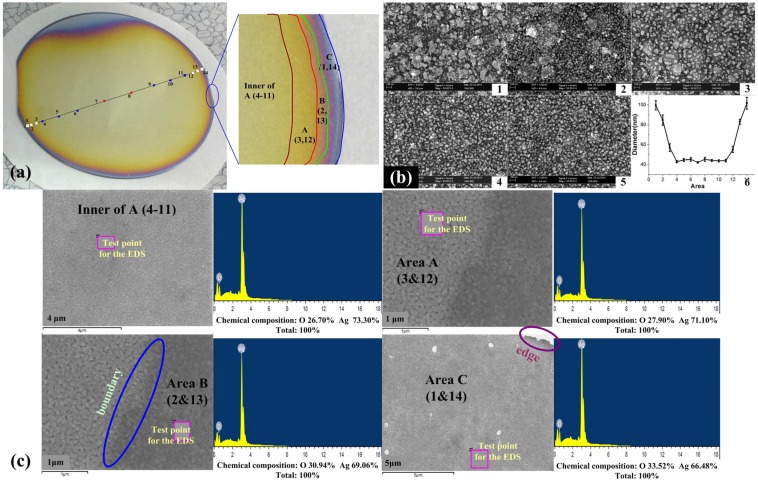
The size of wafer-scale low-dimensional nanomaterilas in the different area. (**a**) the wafer-scale nanomaterials fabricated by the Oxygen atmosphere; (**b**) the scanning electron microscope (SEM) image for the nanoparticles; (**c**) the corresponding energy spectrum.

Figure 4 shows the wafer-scale low-dimensional ordered structures fabricated by the oxygen atmosphere. The wafer have been divided into four areas and been numbered as: inner of A (4 to 11), A (3, 12), B (2, 13) and C (1, 14) respectively. This multi-area composition assessment to test the uniformity of the wafer-scale manufacturing and the reliability of crafts. In our experiment, the sizes of nanoparticles have been calculated by the SmileView software.

As shown in Figure 4b,c, from the central area 7 to the edge area 1 and 14, the nanoparticles size was about 40 nm from the 7 to 4 and 7 to 11 and increased to 100 nm from the area 4 to 1 and 11 to 14. The corresponding energy spectrum shows the similar changes of oxygen content in the corresponding area to prove the same results. The reason is that the air stream is uniformity concentration in the center of wafer and become gathering at the edge. By statistical analysis and effective computing, the uniformity of the wafer structure was larger than 80%.

### 2.4. Surface Raman Enhancement and Surface Fluorescence Enhancement

The SERS activity of the silver material was investigated using crystal violet as a molecular probe and the density is 10^−6^ M in the spectral range of 200–1800 cm^−1^. There are about four Raman characteristic lines for the CV molecule. There exist various bands for CV at around 911 cm^−1^ attributed to benzene ring vibrating, 1176 cm^−1^ for C–H bending vibration; 1370 cm^−1^ for C_center_–C stretching vibration, while the band at 1587 cm^−1^ is related to C–O stretching [17]. As reported in the literature, the final electromagnetic enhancement strongly depends on the size, shape, and distribution of the metallic nanostructure [18]. The mechanism of the surface-enhanced Raman scattering is of an electromagnetic nature and it is based on localized surface plasmons (LSP) [19]. When a surface plasmon is confined to a particle with a size comparable to the wavelength of light, that is, a nanoparticle, the particle free electrons participate in a collective oscillation; this phenomenon, termed “local surface plasmon” [20], enhances significantly the molecular Raman scattering response.

**Figure 5:**
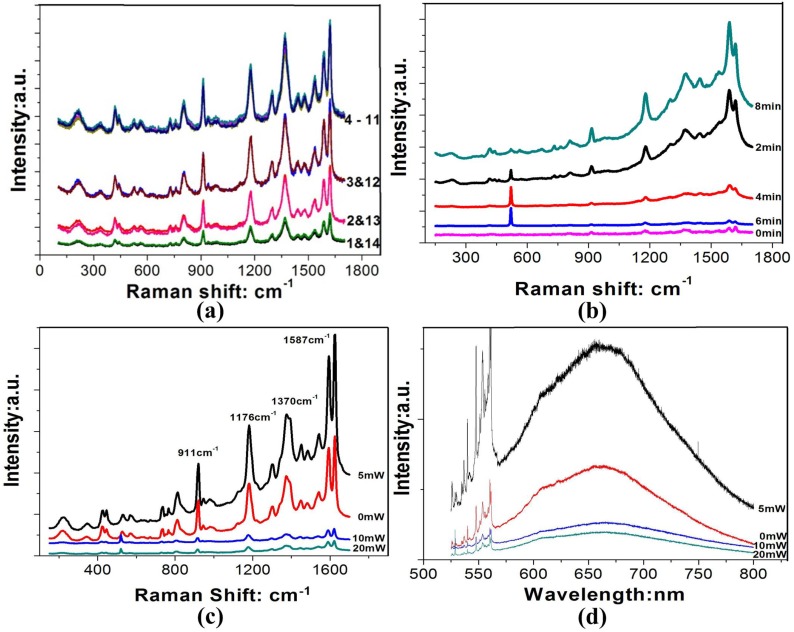
Raman and photoluminescence spectrum of the silver nanomaterials. (**a**) At the different area of the wafer-scale nanomaterials; (**b**) Different treatment time by the oxygen atmosphere; (**c**,**d**) Raman and PL under different power by the argon atmosphere.

As shown in Figure 5a, similar to the section 2.3, the Raman intensity is steady at the area of 4–11, and reduces in turn from the area 4 to 1 and 11 to 14. This proves the uniform of the wafer-scale nanomaterials fabricated by our reporting methods.

As shown in Figure 5b, the intensity of the SERS peaks initially decreased and then increased with the increase of the treatment time by the oxygen atmosphere. From the Figure 5b, there is a sharp Raman peak at about 520 cm^−1^ which is the Raman characteristic peak for single crystal Si. In our experiment, the nano-film were deposited on the Si wafer. As shown in Figure 2b, the nanoparticles were reuniting and growing bigger and bigger with increasing the treatment time. And the more and more holes have been leaving in the films, that is, more and more area would be the Si wafer. So, as shown in Figure 5b, with the treatment time increasing from the 2 min to 6 min, the Raman signal for the single crystal Si increased and Raman signal for the CV decreased. However, with the treatment time of 8 min, the Raman signal for the CV increased. The reason is that the density of the holes structure increases with the plasma treatment time increased. Such nano-scale holes may act as hot spots to enhance the local electromagnetic fields and transfer more energy to the molecule or electron and enhance the Raman signal [21].

As shown in Figure 5c,d, due to the nano-film have been formed the nanoparticles, Raman and PL signal increased with the treatment power from the 0 mW to 5 mW, but the enhance factor were not too high. Meanwhile, when the plasma power increases by the argon atmosphere, the mean particle size decreases and the shape is not irregular, leading to a decrease of the SERS and SEF intensity. So, optimizing the plasma-induced parameters can enhance the sensitivity of the biochemical sensors and wafer-scale fabricating.

## 3. Experimental

P-type silicon wafers were used as the substrate. The purity of the silver target was 99.99%. Solutions with 10^−5^ mol/L CV were prepared. All the experiments were performed in a clean room with a constant temperature of 20 °C and constant relative humidity of 60%.

The Ag films were deposited in a high vacuum system equipped with a DC magnetron sputtering source (Qprep500, Mantis, England, UK). The DC magnetron sputtering was performed under the following conditions: DC power of 45 W; Ar flow rate of 30 sccm; chamber pressure of 7.5 × 10^−3^ Torr. An inductively coupled plasma etching system (System 100-ICP) from Oxford Instruments Plasma Technology (OIPT) was employed for the plasma treatments.

The morphology and energy spectrum of the nanoparticles were characterized by scanning electron microscopy (SEM, SUPRA 55 SAPPHIRE, Carl Zeiss AG, Jena, Germany). The nanoparticle size and density calculations were performed with the assistance of the Smile View software.

CV in water was used as the probe molecule. The SERS and photoluminescence (PL) characterizations were performed after immersion of the samples in aqueous solutions and subsequent drying prior to the acquisition of the spectra. The samples were excited using a 514.5 nm laser line from the Renishaw Raman Microscope System Invia with an excitation power beam of 5 mW.

## 4. Conclusions

A method to fabricate wafer-scale silver nanomaterials using a plasma-induced technique is demonstrated. The silver films were synthesized on a p-doped silicon substrate using a DC magnetron sputtering process. Silver nanomaterials of different sizes and shapes were produced by tuning the synthesis parameters such as the plasma treatment time and gas composition. From the structural characterization of the nanoparticle films, it is concluded that this method can be employed to fabricate several other nanomaterials, such as metals and semiconductors, for applications as sensors.
